# Case Report: Mercury exposure and anti-LGI1/Caspr2 autoimmune encephalitis: a case series and literature review on the paradoxical worsening after chelation therapy

**DOI:** 10.3389/fimmu.2026.1855642

**Published:** 2026-07-21

**Authors:** Chao Huang, Tao Chen, Ge Tan, Junming Li, Ling Liu

**Affiliations:** 1 Department of Neurology, West China Hospital, Sichuan University, Chengdu, China; 2 Department of Geriatric Medicine and Neurology, West China School of Public Health / West China Fourth Hospital, Sichuan University, Chengdu, China; 3 Department of Radiology, West China School of Public Health and West China Fourth Hospital, Sichuan University, Chengdu, China; 4 Department of Psychiatry, West China Hospital, Sichuan University, Chengdu, China

**Keywords:** autoimmune encephalitis, case series, Caspr2, chelation therapy, LGI1, mercury

## Abstract

**Background:**

Chronic mercury exposure may be a potential risk factor for anti-LGI1/Caspr2 autoimmune encephalitis (AE). The effect of chelation therapy on coexisting AE is unknown.

**Methods:**

We report three patients from West China Fourth Hospital, Sichuan University (September–November 2025) with confirmed chronic mercury exposure, anti-LGI1/Caspr2 antibody positivity, and DMPS chelation. Clinical course, antibody titers, and treatment response were analyzed.

**Results:**

Within 1-3 days of chelation initiation, all three patients developed acute neuropsychiatric deterioration, a phenomenon not previously documented. Each patient fully recovered with prompt immunotherapy, with favorable long-term outcomes (mRS 0–2). This observation appears to contrast with experimental animal models where chelators attenuate rather than exacerbate autoimmune neuroinflammation.

**Conclusion:**

This series suggests that chelation therapy may be temporally associated with acute worsening in this specific population, highlighting a potential therapeutic dilemma: the standard treatment for mercury may, in some cases, be associated with exacerbation of the autoimmune process that may be related to its use. Screening for occult heavy metal exposure may be considered in unexplained AE, and immunotherapy should be initiated promptly if deterioration occurs.

## 1 Introduction

Mercury exposure is an established but frequently overlooked environmental risk factor for autoimmune phenomena. Its potential role in autoimmune encephalitis (AE)—particularly in anti-LGI1 and anti-Caspr2 antibody-associated syndromes—remains poorly defined, with only scattered case reports in the literature (1–4). Even less understood is the paradoxical effect of chelation therapy, which has been sporadically implicated in neurological deterioration rather than improvement (3, 5).

Anti-LGI1 encephalitis typically presents with limbic dysfunction, hyponatremia, and faciobrachial dystonic seizures, while anti-Caspr2 antibodies are associated with central and peripheral hyperexcitability, including Morvan’s syndrome (6, 7). Although paraneoplastic and post-infectious triggers are well recognized, environmental toxicants such as mercury have not been systematically investigated in these specific serotypes.

Here we report three patients with confirmed chronic mercury exposure and anti-LGI1/Caspr2 encephalitis—one positive for anti-LGI1, one for anti-Caspr2, and one dual-positive—all of whom exhibited acute neuropsychiatric worsening immediately following chelation therapy. Through detailed case analysis and literature review, we describe this association and document a consistent phenomenon of exacerbation temporally associated with chelation. These findings suggest that heavy metal screening may be considered in unexplained AE and highlight the need for further evaluation of chelation indications in antibody-positive patients.

## 2 Methods

### 2.1 Literature review

We performed a structured literature review to identify published cases of autoimmune encephalitis (AE) associated with mercury exposure and anti-LGI1/Caspr2 antibodies. Given the rarity of the condition, this review was conducted as a structured search of available case−level evidence with narrative synthesis, informed by PRISMA 2020 principles where applicable. PROSPERO registration was not performed as this review was part of a retrospective case series rather than an independent structured literature review.

PubMed, EMBASE, CNKI, WanFang, and VIP China Science were searched from inception to December 2025 using terms for mercury (“mercury”, “Hg”, “heavy metal”), AE (“limbic encephalitis”, “anti-LGI1”, “Caspr2”), and treatment (“immunotherapy”, “chelation”). The full search string for PubMed is provided in **Supplementary Appendix 1**. No language restrictions were applied. Reference lists of included articles were manually screened.

Two reviewers independently screened titles and abstracts, then reviewed full texts of potentially eligible records. Disagreements were resolved through consensus or consultation with a third reviewer. Studies were included if they met all of the following criteria: (1) confirmed positivity for anti-LGI1 and/or anti-Caspr2 antibodies in serum or cerebrospinal fluid; (2) documented history of mercury exposure; (3) sufficient clinical data to extract presentation, treatment, and outcome. Reviews, commentaries, duplicate publications, and studies lacking antibody confirmation or clear exposure history were excluded. Studies with positive antibody without encephalitic symptoms, or with encephalitic manifestations without antibody testing, were also excluded.

All records were imported into EndNote X9, and duplicates were removed by comparing titles, authors, journal, and publication year. The search yielded 39 records across five databases. After removing 18 duplicates, 21 records were screened. Eight were excluded by title/abstract (not original studies, not related to mercury, or not relevant to anti−LGI1/Caspr2). One full−text article could not be retrieved. Of 12 full−text articles assessed, 8 were excluded: 5 with encephalitic manifestations lacking antibody testing, and 3 with positive antibody without encephalitic symptoms. Ultimately, four articles reporting 13 unique patients met the inclusion criteria. The clinical characteristics and quality assessment of these cases are summarized in **Supplementary Table 1**, and the literature selection process is presented in **Figure 1**.

**Figure 1:**
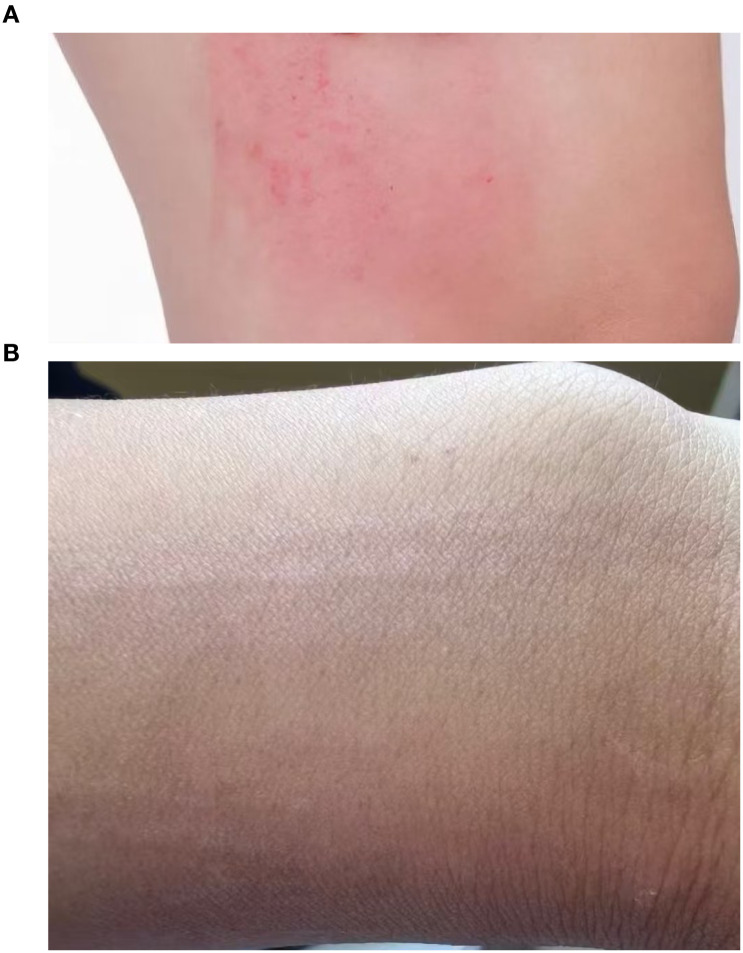
Cutaneous erythematous lesions in Case 2 patient. **(A)** Diffuse patchy erythema with scattered punctate lesions on the trunk skin; **(B)** Mild localized faint erythema on the limb skin.

### 2.2 Standard protocol approvals, registrations, and patient consents

This retrospective case series was conducted in accordance with the ethical standards of the institutional research committee. Formal ethical approval was waived due to the anonymized nature of the clinical data and the absence of any identifiable patient information. Written informed consent for publication of their clinical details was obtained from all patients or their legal guardians. This case series was prepared in accordance with the CARE (Case report) guidelines, and the completed CARE checklist is provided as **Supplementary Material**.

## 3 Case reports

### 3.1 Case 1

#### 3.1.1 History

A female patient in her 60s presented with an 8−day history of significant memory decline accompanied by insomnia, hyperhidrosis, and palpitations. On admission, her Activities of Daily Living (ADL) score was 26, and her modified Rankin Scale (mRS) score was 3. Her medical history was notable only for hypertension. Further history obtained from family members revealed that symptoms had actually begun two months earlier, presenting as tremor while holding objects, arthralgia, and low−grade fever.

#### 3.1.2 Physical and laboratory examinations

Physical examination on admission revealed an anxious but alert and fully oriented patient with stable vital signs. Neurological examination showed slowed speech, impaired short-term memory, and bilateral hand tremor when holding utensils, with intact cranial nerves, normal muscle strength and tone, and preserved sensation and reflexes, though finger-to-nose and heel-to-shin tests were slightly unsteady.

Initial laboratory studies, including comprehensive immune serology, tumor markers, and endocrine profiles, as well as brain MRI, were unremarkable. Lumbar puncture showed cerebrospinal fluid (CSF) protein of 0.53 g/L (normal range 0.15–0.45 g/L), nucleated cell count of 10 x10^6^/L with 96.2% mononuclear cells, and glucose of 2.9 mmol/L (reference range 2.5–4.5 mmol/L). CSF Gram staining, acid−fast bacilli staining, and fungal smear were all negative. The patient was initially diagnosed with viral meningoencephalitis and received acyclovir (500 mg intravenously every 8 hours). After 10 days, her neurological symptoms showed no improvement. Given that the patient resided in a mining area, environmental exposure risks were considered. Heavy metal screening was performed and revealed elevated mercury levels (blood mercury: 24.2 μg/L, reference <5 μg/L; urinary mercury: 113 μmol/mol creatinine, reference <5 μmol/mol creatinine) (**Table 1**). Admission inflammatory markers and CSF studies suggested blood−brain barrier dysfunction and intrathecal immunoglobulin synthesis. During deterioration, white blood cell and lymphocyte counts increased, indicating an inflammatory response, which normalized before discharge (**Supplementary Table 2** for details).

**Table 1:** Clinical characteristics, treatment response, and prognosis of patients.

Characteristic	Case 1	Case 2	Case 3
Demographics			
Age group	60s	40s	50s
Exposure source	Environmental (mining area)	Iatrogenic (suspected unregulated medication use)	Occupational (metallurgy)
Estimated exposure duration	8 years	2 months	2 years
Onset symptoms	Memory decline, insomnia, tremor, arthralgia	Dizziness, headache, memory decline	Generalized pain, fatigue, speech disturbance
Pre-treatment mRS score	3	3	4
Pre-treatment ADL score	26	34	28
Pre-treatment laboratory findings			
Blood mercury (μg/L) [Ref <7.8]	24.2	4.68	14.7
Urine mercury (μmol/mol creatinine) [Ref<2.25]	113	35.5	174.6
Chelation regimen	IV DMPS 250 mg/day, 3 days on/4 days off, 2 cycles	IV DMPS 250 mg/day, 3 days on/4 days off, 2 cycles	IV DMPS 375 mg/day, 3 days on/4 days off, 2 cycles
Onset of exacerbation	Day 1	Day 3	Day 1
Worsening Symptoms	Staring spells, seizures, encephalopathy	Seizures (refractory), encephalopathy, rigidity, hallucinations	Encephalopathy, lethargy, myokymia, agitation
Laboratory findings during treatment			
Blood mercury (μg/L) [normal <7.8]	23.1	2.87	9.35
Urine mercury (μmol/mol creatinine) [normal <2.25]	197.4	438.6	197.8
Autoantibodies (CSF/serum)	CSF/serum	CSF/serum	CSF/serum
anti-LGI1 IgG	1:3.2/1:10	1:10/–	Negative
anti-CASPR2 IgG	–/1:10	Negative	1:1/1:320
Immunotherapy Received	IVMP → oral prednisone, valproate	IVIG (0.4 g/kg/day) for 5 days → prednisone	IVMP (worsening) → TPE
Post-treatment laboratory findings			
Blood mercury (μg/L) [normal <7.8]	20.1	3.62	3.42
Urine mercury (μmol/mol creatinine) [normal <2.25]	45.2	6.98	6.51
Outcome (follow-up)			
anti-LGI1 IgG (serum)	Negative	Negative	Negative
anti-CASPR2 IgG (serum)	Negative	Negative	1:32
Blood mercury (μg/L) [normal <7.8]	Normal	3.05	3.89
Urine mercury (μmol/mol creatinine) [normal <2.25]	Normal	1.73	9.23
mRS score	1	0	2
ADL score	17	14	17

ADL, Activities of Daily Living; CSF, cerebrospinal fluid; DMPS, sodium 2,3-dimercapto-1-propanesulfonate; GTCS, generalised tonic−clonic seizures; IV, intravenous; IVIG, intravenous immunoglobulin; IVMP, intravenous methylprednisolone; mRS, modified Rankin Scale; TPE, therapeutic plasma exchange. Reference ranges are shown in parentheses.

#### 3.1.3 Chelation therapy and neurological deterioration

Chelation therapy with intravenous sodium 2,3-dimercapto-1-propanesulfonate (DMPS) (250 mg daily, 3 days on and 4 days off, for 2 cycles) was initiated. On day 1 of treatment, the patient developed episodic staring spells and psychomotor slowing, followed by sudden loss of consciousness accompanied by cluster seizures. Neurological evaluation and a repeat lumbar puncture were performed. Autoimmune encephalitis antibody testing was performed using a cell−based assay and was positive for anti-LGI1 IgG (CSF titer 1:3.2, serum titer 1:10) and anti-CASPR2 IgG (serum titer 1:10). Immunotherapy was initiated concurrently.

#### 3.1.4 Treatment and follow-up

The patient received intravenous methylprednisolone (IVMP) (1 g daily for 3 days, then 0.5 g daily for 3 days), followed by oral prednisone (1 mg/kg/day) and valproate (0.5 g twice daily). Within two weeks, memory improved and no further seizures or confusion occurred. Blood and urinary mercury levels normalized. At the 4-month follow-up, serum anti−LGI1 and anti−CASPR2 antibodies were negative (CSF was not retested). Her mRS score had improved to 1 and ADL score to 17.

### 3.2 Case 2

#### 3.2.1 History

A 40-year-old female was admitted with a 20-day history of dizziness and headache, followed by progressive memory decline over 15 days. On admission, the ADL score was 34, and mRS score was 3. During hospitalization, the patient developed visual hallucinations, severe insomnia, progressive cognitive deterioration, and generalized pruritus.

#### 3.2.2 Physical and laboratory examinations

On arrival, the patient was alert and cooperative, though her speech content was partially tangential and she appeared anxious. Vital signs were stable. Multiple excoriations were observed on the skin, without rash or petechiae. No superficial lymphadenopathy was palpable, and cardiopulmonary and abdominal examinations were unremarkable. Neurological examination showed fluent speech with partially irrelevant content and markedly reduced short−term memory. Cranial nerves were intact. Muscle tone was normal, with no atrophy or fasciculations. Deep sensation was intact, but hyperalgesia was noted. Tendon reflexes were slightly brisk. Coordination was normal, and no pathological reflexes were elicited.

Routine laboratory studies and brain MRI were unremarkable (**Figure 2**). Electroencephalogram (EEG) showed significantly increased generalized theta and delta slow waves over all leads, with loss of the normal posterior dominant alpha rhythm (**Figure 1**). Further inquiry into potential exposure history was pursued. Family members subsequently reviewed the patient’s mobile phone and discovered that the patient had purchased unregulated substances online for the purpose of menstrual regulation in the two months prior to admission. Toxicology screening was therefore performed and revealed elevated urinary mercury (35.5 μmol/mol Cr; reference range 0–2.25) and a high-normal blood mercury level (4.68 μg/L; reference range 0–7.8).

**Figure 2:**
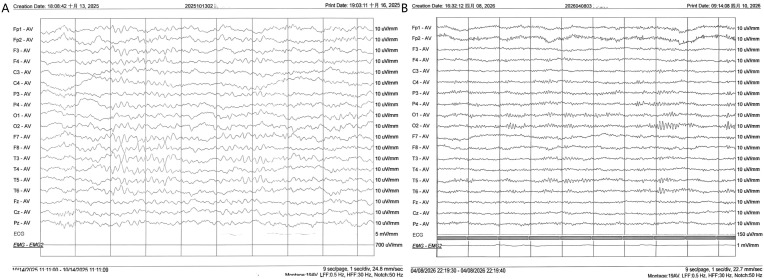
Electroencephalogram (EEG) findings in Case 2 before and after treatment. **(A)** Pretreatment EEG showing diffuse generalized theta and delta slow waves across all electrodes with loss of the normal posterior dominant alpha rhythm; **(B)** Posttreatment EEG showing normalization of background activity with reappearance of the posterior dominant alpha rhythm and no epileptiform discharges.

#### 3.2.3 Chelation therapy and neurological deterioration

Intravenous DMPS chelation therapy (250 mg daily, 3 days on and 4 days off, for 2 cycles) was initiated. On the third day of treatment, the patient developed neuropsychiatric symptoms, including grasping at clothes, unresponsiveness, bradyphrenia, and rigidity of both upper limbs. Each episode lasted approximately one minute, followed by complete recovery of consciousness but subsequent amnesia for the event. These episodes occurred in clusters and eventually progressed to generalized tonic-clonic seizures. Seizures were poorly controlled by conventional first- and second-line anticonvulsants (including diazepam, phenobarbital, and valproate sodium) in the general ward. The patient was then transferred to the intensive care unit, where seizure termination was achieved with midazolam. During this period, a lumbar puncture was performed. Anti-LGI1 IgG antibody was detected in the cerebrospinal fluid (CSF titer 1:10) using a live cell-based assay.

#### 3.2.4 Treatment and follow-up

The patient subsequently received intravenous immunoglobulin followed by a tapering course of prednisone. Within 3 weeks, seizures, neuropsychiatric symptoms, and pruritus completely resolved. Mercury levels declined (urinary mercury: 2.65 μmol/mol Cr; blood mercury: 3.05 μg/L). At the 4-month follow-up, the patient was asymptomatic, with normalization of mercury levels and negative anti-LGI1 antibodies. The ADL score was 14, and the mRS score was 0.

### 3.3 Case 3

#### 3.3.1 History

A patient in their 50s presented with a 3-month history of generalized pain and a 1-day history of fatigue accompanied by speech disturbance. Prior evaluations performed at a local hospital, including cerebrospinal fluid studies, anti-ganglioside antibody testing, and neuroimaging (**Figure 3**), were unremarkable.

**Figure 3:**
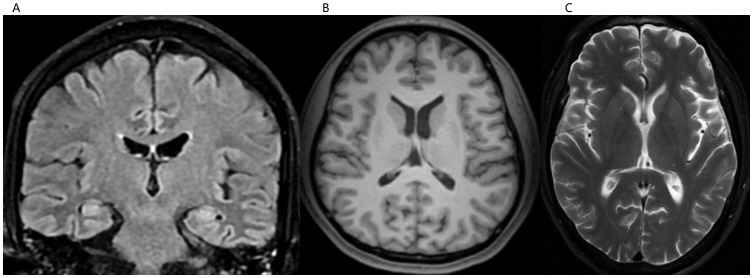
Representative brain MRI scans of Case 2. **(A)** Coronal FLAIR sequence; **(B)** Axial T1-weighted image (T1WI); **(C)** Axial T2-weighted image (T2WI). No abnormal signal intensities were identified on any sequence, and all three patients presented unremarkable brain MRI findings.

#### 3.3.2 Physical and laboratory examinations

On admission, the patient was awake and fully oriented, with stable vital signs and normal skin and mucous membranes. Neurological examination showed mildly non−fluent speech with normal memory, orientation, and comprehension. Cranial nerves were intact. Sensation and muscle tone were normal. Muscle strength was 4/5 in both lower limbs, with spontaneous fasciculations observed in the calves and no significant muscle atrophy. Tendon reflexes were brisk. No pathological reflexes were elicited. The remainder of the general and neurological examination was unremarkable.

Given an occupational history in metallurgy over the preceding two years, heavy metal screening was performed and demonstrated elevated mercury levels (blood mercury: 14.7 μg/L; urinary mercury: 372 μmol/mol Cr).

#### 3.3.3 Chelation therapy and neurological deterioration

Intravenous chelation therapy with DMPS (375 mg daily, 3 days on and 4 days off, for 2 cycles) was initiated. On the first day after chelation, the patient developed neuropsychiatric symptoms including incoherent speech, lethargy, urinary frequency, and lower limb myokymia. A repeat lumbar puncture was performed. The cerebrospinal fluid (CSF) was clear and transparent, with a nucleated cell count of 12 ×10^6^/L, a mononuclear cell proportion of 74.2%, CSF protein of 0.3 g/L, and CSF glucose of 4.5 mmol/L, all within normal ranges. CSF testing using a cell−based assay was positive for anti-CASPR2 IgG (CSF titer 1:1; serum titer 1:320). The patient then received IVMP (1 g). After one day of treatment, the condition acutely worsened, manifesting as severe insomnia, agitation, and progressive speech disturbance.

#### 3.3.4 Treatment and follow-up

Steroids were discontinued, and therapeutic plasma exchange (30 mL/kg/day) was instituted. The patient received an initial 3−day course, followed by a 5−day course one week later. Within 2 weeks, pain, dysarthria, and myokymia completely resolved. At the 3−month follow−up, the patient had an ADL score of 17 and an mRS score of 2, with normalized mercury levels. Serum anti−CASPR2 antibody titer was 1:32 at follow−up, cerebrospinal fluid was not retested.

## 4 Discussion

Managing patients with coexisting chronic mercury poisoning and anti-LGI1/Caspr2 antibody-positive AE presents a diagnostic and therapeutic dilemma. Although sporadic cases of this co-occurrence have been reported, the effect of standard chelation therapy on the clinical course of AE is unknown. We describe three patients with confirmed chronic mercury exposure and anti-LGI1/Caspr2 AE, all of whom developed acute, stereotyped neuropsychiatric deterioration within 1–3 days of initiating DMPS chelation therapy—a paradoxical worsening not previously documented. MRI was normal in all three patients. EEG was normal in Cases 1 and 3, Case 2 showed EEG abnormalities at admission, which normalized after immunotherapy—further supporting the immune−mediated nature of the encephalopathy. This series provides three novel insights: (i) supporting the mercury -VGKC complex association; (ii) suggesting a temporal association with acute clinical worsening; and (iii) raising a therapeutic dilemma that warrants further investigation.

### 4.1 Mercury-associated anti-LGI1/Caspr2 encephalitis: an under-recognized entity

Chronic mercury exposure is a well-established cause of neurological and renal toxicity, yet its role as a trigger for anti−LGI1/Caspr2 encephalitis remains poorly quantified. A systematic literature search of PubMed, Embase, and Web of Science (up to December 2025) identified only isolated case reports (1–3, 8).

To date, fewer than 50 cases of mercury-induced VGKC-complex autoimmunity have been reported globally, predominantly from China and the United States (2, 3, 8–12). A 2020 review identified 41 cases of mercury-induced Morvan syndrome (3), since then, additional cases—including the youngest documented patient (14 months) and a case with concurrent membranous nephropathy—have been reported (8, 9). The clustering of three cases at a single tertiary center over three months suggests this association may be under-recognized, though whether it is truly rare remains uncertain given the lack of systematic screening data.

This series provides a systematic description of mercury exposure specifically associated with anti-LGI1 and/or anti-Caspr2 antibody-positive encephalitis, and documents a consistent, temporally associated exacerbation following chelation.

An important alternative interpretation is that the observed antibody positivity could be incidental or secondary. We have carefully considered these possibilities. Incidental positivity is unlikely given the convergent evidence supporting antibody pathogenicity: clinical phenotypes matching known antibody spectra, CSF inflammation, temporal correlation between titers and clinical course, and immunotherapy response. Secondary positivity following viral infection has been reported, but none of our patients had a preceding infectious illness, and CSF viral studies were negative. All patients responded to immunotherapy, with antibody titers declining in parallel with clinical improvement—findings more consistent with a primary pathogenic role. Nevertheless, without large−scale epidemiological data, we cannot statistically exclude chance coexistence, and an occult trigger cannot be completely ruled out. While the evidence favors a pathogenic role, causality cannot be definitively established from this small series.

The differential diagnosis was systematically considered. Viral encephalitis was excluded by negative CSF PCR and lack of response to acyclovir. Paraneoplastic causes were ruled out by comprehensive tumor screening. Mercury neurotoxicity was distinguished from AE by CSF inflammation, specific antibody detection, and immunotherapy response. Morvan syndrome was considered in the two CASPR2−positive patients but neither met full diagnostic criteria: Case 1 lacked myokymia and REM sleep behavior disorder. Case 3 had clinical myokymia, but electromyography showed no definite abnormalities, and autonomic dysfunction was confined to isolated urinary frequency without multisystem involvement. Metabolic and non−autoimmune seizure disorders were excluded by normal laboratory findings and poor response to conventional anticonvulsants.

These observations suggest that heavy metal screening may be considered in the diagnostic workup of unexplained AE, particularly in patients with occupational or environmental exposure.

### 4.2 The paradox of chelation-induced worsening: mechanisms and hypotheses

The acute neuropsychiatric deterioration observed in all three patients within 1–3 days of initiating DMPS chelation contrast with experimental studies showing that chelators can suppress autoimmune neuroinflammation. In the cuprizone model of toxic demyelination, feeding with this copper chelator rendered mice resistant to subsequent induction of experimental allergic encephalomyelitis (EAE), associated with impaired T-cell priming and reduced Th1/Th17 responses (13). Similarly, desferrioxamine mesylate (DFOM), an iron chelator, reduces the severity and shortens the duration of EAE (14, 15). These animal studies demonstrate that chelators generally exert immunosuppressive or protective effects in autoimmune CNS models—opposite to the acute exacerbations observed in our patients following DMPS administration.

Notably, DMPS−induced neurological deterioration is not without precedent. A large−sample retrospective case−control study published in 2025 enrolled 277 patients with neurological Wilson’s disease and reported that 24.5% (68/277) developed neurological deterioration following DMPS treatment, primarily presenting with tremors and speech disorders (16). This raises the possibility that the paradoxical worsening following chelation may extend beyond mercury−associated autoimmunity to other neurological conditions.

This paradox demands mechanistic explanation. We propose three non-mutually exclusive hypotheses:

First, immune re-exposure/adjuvant hypothesis. Mercury ions can act as a hapten or adjuvant, modifying self-proteins and breaking immune tolerance (2, 17). Both LGI1 and CASPR2 are disulfide-rich proteins whose conformation may be disrupted by heavy metals such as mercury, potentially exposing novel antigenic epitopes. This could trigger an aberrant immune response through molecular mimicry or epitope spreading. The surge in bioavailable mercury mobilized by chelation could therefore rapidly boost pre-existing LGI1/Caspr2 autoimmunity, precipitating acute symptom exacerbation (18). This mechanism is analogous to cadmium and gold triggering autoimmunity against podocyte antigens (e.g., PLA2R) in membranous nephropathy (19, 20).

This hypothesis is supported by the temporal and biochemical pattern in our patients: (i) urinary mercury was significantly elevated during deterioration, suggesting ongoing mobilization; (ii) the clinical phenotype (seizures/encephalopathy) precisely matched the known antibody specificity; and (iii) the rapid response to immunotherapy indicates immune-mediated rather than direct toxicity. This triad—temporal contiguity, phenotypic specificity, and therapeutic response—is consistent with an antigen-driven immune reactivation mechanism. The heterogeneity in antibody profiles (single or dual positivity) and clinical severity may relate to individual genetic susceptibility, exposure dose, duration, and the chemical form of mercury. Furthermore, the early clinical worsening resembles a Jarisch-Herxheimer-like reaction seen in the treatment of certain infectious diseases, potentially stemming from an inflammatory storm triggered by antigen release or altered immune status following therapeutic intervention. This framing underscores that the clinical scenario is not simply a matter of detoxification, but rather a complex management challenge involving the interaction between the immune system and toxicant exposure.

Second, blood–brain barrier (BBB) modulation. Chelation-induced hemodynamic or oxidative stress may transiently increase BBB permeability, facilitating entry of pre-existing autoantibodies into the CNS. Although DMPS itself does not redistribute mercury to the brain in rodent models (18), the systemic inflammatory and oxidative shifts accompanying rapid metal mobilization could alter BBB integrity (21). This mechanism would explain the stereotyped, acute onset of seizures and encephalopathy—features consistent with antibody-mediated CNS hyperexcitability. Future studies using CSF/serum albumin ratios could test this hypothesis.

Third, a direct neuropharmacological effect of DMPS on neuronal excitability cannot be excluded, though this remains speculative. Structurally related dithiol chelators have been shown to interact with glutamatergic neurotransmission, but DMPS itself did not significantly affect glutamate binding in the same study (22). Whether DMPS modulates neuronal excitability in the mercury−loaded human CNS requires further investigation.

All three patients’ exacerbations were rapidly reversible with prompt immunotherapy (corticosteroids, IVIG, or plasma exchange), and long-term outcomes were favorable (mRS 0–2). This reversibility argues against irreversible structural injury and supports an immune-mediated or functional neuronal hyperexcitability mechanism, rather than direct chelator neurotoxicity.

We acknowledge that the precise mechanism remains speculative, as our observations are clinical and lack corroborative biomarkers (e.g., serial cytokine profiling, BBB integrity imaging, or paired pre-/post-chelation autoantibody titers). Nonetheless, the consistency and reproducibility of the phenomenon across three independent patients suggest a genuine clinical signal. Pending further mechanistic studies, chelation therapy should be approached with caution in this population, with prompt access to immunotherapy if deterioration occurs.

### 4.3 Why mercury exposure preferentially targets the VGKC complex (LGI1/Caspr2)?

A notable feature of our series—and of published cases—is the consistent involvement of LGI1 and/or Caspr2 antibodies. Why does mercury selectively associate with this autoimmunity? We propose several plausible, non-exclusive explanations.

Molecular affinity. Mercury ions (Hg²^+^) have high affinity for thiol (-SH) groups, which are abundant in cysteine-rich protein domains (3). Both LGI1 and Caspr2 contain multiple disulfide bonds and cysteine residues critical for their structure. Mercury binding can modify these proteins or expose cryptic epitopes, creating neoepitopes and breaking immune tolerance (21). This may explain the preferential targeting of VGKC-complex proteins (17).

Genetic susceptibility. Anti-LGI1 encephalitis is strongly associated with HLA-DRB1*07:01 (>90% of patients) (23, 24), while anti-Caspr2 encephalitis associates with HLA-DRB1*11:01 (25). The sibling cases reported by Pérez et al. provide indirect support for this hypothesis: two siblings exposed to the same mercury source both developed Morvan syndrome with anti-LGI1/Caspr2 antibodies, suggesting shared genetic susceptibility (3). Mercury exposure may trigger disease only in genetically predisposed individuals, consistent with a “multiple-hit” model requiring both environmental and genetic factors.

Observed pattern in the literature. Among >50 reported cases of mercury-induced VGKC-complex autoimmunity, most have antibodies against LGI1, Caspr2, or both, rather than other neural autoantigens (3, 9). This contrasts with paraneoplastic or idiopathic AE, where broader antibody spectra are observed, suggesting mercury-associated AE is a pathogenetically distinct subtype.

Anatomical convergence. Mercury tends to accumulate in specific brain regions—including hippocampus, cerebellum, and peripheral nerves—that overlap with high-expression sites of LGI1 and Caspr2 (3, 26, 27). LGI1 is predominantly expressed in the hippocampus and temporal lobe, explaining the limbic encephalitis phenotype (6). Caspr2 is enriched at juxtaparanodes of myelinated axons, explaining peripheral nerve hyperexcitability (9). This anatomical overlap creates a scenario where antigen presentation and immune attack converge on the same structures.

Collectively, these molecular, anatomical, genetic, and epidemiological lines of evidence may help explain why mercury exposure shows such observed specificity for the VGKC complex, and why anti-LGI1/Caspr2 antibodies dominate serologically in these patients.

## 5 Limitations and future directions

This study has several limitations. First, the small sample size (n=3) precludes definitive epidemiological or mechanistic conclusions. In addition, the literature review included only four studies with 13 patients, limiting the epidemiological generalizability of our observations. Second, we did not systematically obtain paired antibody titers at pre−chelation, exacerbation, and recovery time points across all patients, which limits our ability to assess the relationship between antibody dynamics and clinical deterioration. We also did not perform cytokine/chemokine profiling, HLA typing, or immune cell characterization, restricting mechanistic insights. Cognitive function was not assessed using standardized neuropsychological tests, which limits characterization of long−term outcomes in this series. Third, we cannot exclude the possibility that the observed exacerbations reflected the natural history of untreated AE rather than a true chelation effect. However, the temporal stereotypy —all within days of first DMPS dose— and the absence of such rapid fluctuations in non-chelated LGI1/Caspr2 encephalitis cohorts argue against this interpretation. Finally, we did not perform experimental validation in animal models.

Despite these limitations, the consistency and clinical relevance of our observations support: (i) considering routine heavy metal screening in unexplained AE; (ii) close neurological monitoring during chelation therapy in mercury-exposed patients with coexisting autoimmunity; and (iii) a low threshold for initiating immunotherapy if neuropsychiatric deterioration occurs. Larger prospective cohort studies are needed to validate these preliminary observations. Given the exploratory nature of this case series, these findings should be considered hypothesis−generating rather than confirmatory. Future studies should systematically measure serial antibody titers, cytokine/chemokine profiles, HLA typing, and immune cell characterization to further elucidate the mechanisms underlying mercury−associated autoimmunity. Concurrently, multicenter collaborative registries would help define the true prevalence, genetic basis, and optimal management of this under-recognized condition.

## 6 Conclusions

This case series highlights a notable clinical phenomenon at the intersection of mercury toxicity and autoimmune encephalitis. It provides clinical evidence of an association between chronic mercury exposure and anti−LGI1 and/or anti−Caspr2 antibody−positive AE. It also documents a consistent pattern of acute neuropsychiatric deterioration following chelation therapy in this specific population, suggesting a previously underrecognized treatment−related concern. These findings highlight the importance of concurrent screening for both autoimmune antibodies and occult heavy metal exposure in patients with unexplained neuropsychiatric syndromes. Mercury may be not only a potential risk factor for specific autoimmune encephalitides but also a factor that may warrants reconsideration of the risk−benefit balance of its own standard treatment. These observations remain preliminary and require validation in larger prospective cohorts before changes to clinical practice can be considered.

## Data Availability

The original contributions presented in the study are included in the article/**Supplementary Material**. Further inquiries can be directed to the corresponding author.
