# Supplementary material for: Case Report: Mercury exposure and anti-LGI1/Caspr2 autoimmune encephalitis: a case series and literature review on the paradoxical worsening after chelation therapy
Source: Front Immunol. 2026 Jul 21;17:1855642. doi: 10.3389/fimmu.2026.1855642 (PMC13446009; doi:10.3389/fimmu.2026.1855642)
Supplement: Supplementary file 1 [file Table1.docx]

Supplementary Table 1. Summary of clinical characteristics and methodological quality assessment of included studies.

| Case | 1 | 2 | 3 | 4 |
| --- | --- | --- | --- | --- |
| Author/years | Ariel Heller/2024 | Caihong Liu/2025 | Carlos A. Pérez/2020 | G. Gnana shanmugam/2018 |
| Antibody testing method | not reported | not reported | not reported | not reported |
| Mercury exposure history | mercury exposure (details not specified) | suspected occupational exposure | accidental exposure to elemental mercury | unauthorized Siddha medicine |
| Number of cases | 1 | 1 | 2 | 9 |
| Age | 14-Month-old | 55-year-old | 19-year-old female and a 17-year-old male | not reported |
| Gender | female | male | a female and a male | not reported |
| Clinical symptoms | Initial symptoms comprised irritability, fatigue, refusal to bear weight, diarrhea, and regression in motor and language milestones. The condition later progressed to persistent encephalopathy with diffuse motor weakness, sensory loss, hypokinesia, and reduced lower-limb reflexes. | neuropsychiatric disturbances, including incoherent speech and hallucinations | generalized erythematous rash, muscle weakness, paresthesias, limb rigidity, generalized myokymia, insomnia, blurry vision encephalopathy, and seizure-like activity | Encephalopathy |
| Antibodies | LGI1，Caspr2,GAD65,VGKC | LGI1，Caspr2 | LGI1，Caspr2、(GAD65)、N-type and P/Q-type voltage-gated calcium channel (VGCC) | CASPR2 |
| Antibody titer | LGI1(titer not reported), Caspr2 (1:100), GAD 65(0.11nmol/L); VGKC (positive) | titer not reported | titer not reported | titer not reported |
| Treatment modalities | Immunotherapy with corticosteroids and IVIg followed by rituximab | glucocorticoids,  DMPS | intravenous methylprednisolone、IVIg and Rituximab | pregabalin, gabapentin, opioids, and short course of steroids |
| magnetic resonance | normal | normal | normal | normal |
| Electroencephalography | normal | not mentioned | normal | not mentioned |
| Outcome / Prognosis | Full recovery | recovery | Full recovery | improved gradually |
| JBI criteria met (out of 4 assessed domains) | 3/4 | 3/4 | 3/4 | 3/4 |

Abbreviations: DMPS, sodium dimercaptopropane sulfonate; GAD65, glutamic acid decarboxylase-65; IVIg, intravenous immunoglobulin; IVMP, intravenous methylprednisolone; VGCC, voltage-gated calcium channel; VGKC, voltage-gated potassium channel. Note: The JBI-based assessment was performed across four domains: (1) clear documentation of antibody testing methodology; (2) confirmed mercury exposure history; (3) sufficient clinical detail; and (4) availability of follow-up data.

SupplementaryTable2. Clinical timeline of three patients with mercury-associated anti-LGI1/Caspr2 encephalitis.

| Time point | Case 1 (60s/F) | Case 2 (40s/F) | Case 3 (50s/M) |
| --- | --- | --- | --- |
| Symptoms onset | 2 months prior: memory decline, insomnia, tremor, arthralgia | 20 days prior: dizziness, headache; 15 days: memory decline | 3 months prior: generalized pain; 1 day: fatigue, speech disturbance |
| Exposure source | Environmental (mining area) | Iatrogenic (suspected unregulated medication use) | Occupational (metallurgy) |
| Exposure duration | 8 years | 2 months | 2 years |
| Admission (Day 0) | mRS 3, ADL 26 | mRS 3, ADL 34 | mRS 4, ADL 28 |
| Inflammatory markers on admission | WBC4.37x10^9^/L(normal); hs-CRP<4mg/L(normal) | Lymphocytes 0.95x10^9^/L (↓); WBC4.79x10^9^/L(normal); hs-CRP 0.20mg/L(normal) | hs-CRP 26.78mg/L (↑); Neutrophils 76.8% (↑) |
| Mercury diagnosis | Blood Hg 24.2 μg/L; Urine Hg 113 μmol/mol Cr | Blood Hg 4.68 μg/L; Urine Hg 35.5 μmol/mol Cr | Blood Hg 14.7 μg/L; Urine Hg 174.6 μmol/mol Cr |
| Antibody confirmation | LGI1 (CSF 1:3.2, serum 1:10); Caspr2 (serum 1:10) | LGI1 (CSF 1:10) | Caspr2 (CSF 1:1, serum 1:320) |
| EEG | normal | abnormal(pre-treatment); normalized after immunotherapy | normal |
| MRI | normal | normal | normal |
| DMPS chelation started | Day 0 | Day 0 | Day 0 |
| Chelation regimen | IV DMPS 250 mg/day, 3 days on/4 days off, 2 cycles | IV DMPS 250 mg/day, 3 days on/4 days off, 2 cycles | IV DMPS 375 mg/day, 3 days on/4 days off, 2 cycles |
| Acute deterioration onset | Day 1: staring spells, seizures, encephalopathy | Day 3: refractory seizures, encephalopathy, rigidity | Day 1: encephalopathy, lethargy, myokymia, agitation |
| Inflammatory markers during deterioration | WBC8.06x10^9^/L(normal); Lymphocytes 4.44x10^9^/L (↑); CSF albumin 530mg/L (↑), nucleated cell count of 10x10^6^/L (↑) | WBC11.94x10^9^/L (↑); Lymphocytes 0.53x10^9^/L (↓); CSF albumin 229mg/L(normal), nucleated cell count of 1x10^6^/L(normal) | hs-CRP 66.19mg/L (↑); WBC12.27x10^9^/L (↑); Lymphocytes 0.82x10^9^/L (↓); CSF IgG 0.067g/L (↑), CSF albumin 0.286g/L (↑), CSF albumin 300mg/L(normal), nucleated cell count of 12x10^6^/L (↑) |
| Immunotherapy initiated | Day 2: IVMP → oral prednisone | Day 4: IVIG → prednisone | Day 2: IVMP (worsening) → TPE |
| Clinical recovery | 2 weeks: memory improved, seizures resolved | 3 weeks: seizures, neuropsychiatric symptoms resolved | 2 weeks: pain, dysarthria, myokymia resolved |
| Inflammatory markers pre-discharge | WBC, lymphocytes, and hs-CRP normalized | WBC, lymphocytes, and hs-CRP normalized | hs-CRP 4.72mg/L(normal), WBC6.91x10^9^/L (↑); Lymphocytes 1.27x10^9^/L(normal) |
| Follow-up mRS/ADL | mRS 1, ADL 17 (4 months) | mRS 0, ADL 14 (4 months) | mRS 2, ADL 17 (3 months) |
| Antibody status at follow-up | Negative(serum) | Negative (serum) | anti-CASPR2 IgG (serum): 1:32 |

Abbreviations: hs-CRP, high‑sensitivity C‑reactive protein; ADL, Activities of Daily Living; CSF, cerebrospinal fluid; MRI, Magnetic Resonance Imaging; EEG, Electroencephalogram; DMPS, sodium 2,3-dimercapto-1-propanesulfonate; IVIG, intravenous immunoglobulin; IVMP, intravenous methylprednisolone; mRS, modified Rankin Scale; TPE, therapeutic plasma exchange.
