# Supplementary material for: Case Report: Mercury exposure and anti-LGI1/Caspr2 autoimmune encephalitis: a case series and literature review on the paradoxical worsening after chelation therapy
Source: Front Immunol. 2026 Jul 21;17:1855642. doi: 10.3389/fimmu.2026.1855642 (PMC13446009; doi:10.3389/fimmu.2026.1855642)
Supplement: Supplementary file 2 [file Table2.docx]

(("mercury"[MeSH Terms] OR "mercury"[Title/Abstract] OR "Hg"[Title/Abstract] OR "heavy metal"[Title/Abstract]) AND ("autoimmune encephalitis"[MeSH Terms] OR "autoimmune encephalitis"[Title/Abstract] OR "limbic encephalitis"[Title/Abstract] OR "anti-LGI1"[Title/Abstract] OR "Caspr2"[Title/Abstract] OR "VGKC complex"[Title/Abstract]) AND ("immunotherapy"[MeSH Terms] OR "immunotherapy"[Title/Abstract] OR "chelation"[Title/Abstract] OR "DMPS"[Title/Abstract] OR "dimercaprol"[Title/Abstract])) AND (english[Filter])
